# Diabetic retinopathy among Brazilian Xavante Indians

**DOI:** 10.1186/s13098-018-0348-z

**Published:** 2018-06-13

**Authors:** Carlos Gustavo M. G. Lima, Laercio Joel Franco, Amaury L. Dal Fabbro, Edson Z. Martinez, João Paulo Botelho Veira-Filho, Alexandre A. C. M. Ventura, Leonardo Prevelato, Antonio Augusto V. Cruz

**Affiliations:** 1 Department of Ophthalmology, Otorhinolaryngology and Head and Neck Surgery, Ribeirão Preto Medical School, University of São Paulo, Av. Bandeirantes, 3900, Ribeirão Preto, SP 14049-900 Brazil; 2 Department of Social Medicine, Ribeirão Preto Medical School, University of São Paulo, Ribeirão Preto, SP 14049-900 Brazil; 3 Division of Endocrinology, São Paulo Medical School, Federal University of São Paulo, São Paulo, SP 04038-001 Brazil; 4 Department of Ophthalmology, Fernando Ventura Eye Institute, Recife, PE 52010-140 Brazil

**Keywords:** Diabetes mellitus, Diabetic retinopathy, Indians, South American

## Abstract

**Background:**

To describe the frequency of diabetic retinopathy (DR) and its associated variables in Brazilian Xavante Indians.

**Methods:**

A population-based survey carried out in two Xavante Reservations between 2008 and 2012, included 948 Indians aged 20 years or more, identified 246 individuals with type 2 diabetes. A non-probabilistic cluster sample of 140 diabetic individuals were submitted to ophthalmologic examination. Due to operational conditions and to optimize the field work, only the larger Xavante villages were included. Ophthalmologic examinations were performed during one trip to each reservation, in their villages and consisted of measurement visual acuity, anterior segment biomicroscopy, applanation tonometry, and direct and indirect ophthalmoscopy.

**Results:**

The frequency of DR was 19.3%, distributed as follows: mild non-proliferative retinopathy in nine (33.3%) subjects, moderate in nine (33.3%), severe in six (22.3%), very severe in two (7.4%), and high-risk proliferative DR in one (3.7%). The occurrence of DR was higher among those with a longer duration of diabetes, higher levels of glycated hemoglobin (HbA1c) and fasting glucose, papillary excavation ≥ 0.5, and among individuals in older age group. Using the log-binomial regression model, diabetes duration > 24 months and HbA1c ≥ 6.5% were significantly associated with the occurrence of DR.

**Conclusions:**

The presence of DR (19.3%) in Xavante Indians is an alert for health care providers for this population, since diabetes is a new disease among them. Its association with disease duration, high levels of HbA1c and blood glucose calls attention for the necessity of more actions to improve diabetes control in this recently contacted ethnic group that needs particular attention.

## Background

The prevalence of type 2 diabetes mellitus (T2DM) is increasing worldwide, and in some areas, the disease has reached epidemic status [1]. A distinctive characteristic of the epidemiology of this disease is the wide geographic and ethno-cultural variations in its prevalence. Indigenous people seem to be disproportionately affected, probably due to genetic susceptibility and lifestyle changes [2, 3]. The highest rates of diabetes were found in natives of Nauru, a Pacific Island, and in Pima Indians in the United States, where the prevalence was as high as 50% [1].

Xavante is an indigenous population living in Mato Grosso State, central Brazil, belong to the Macro-Jê linguistic group, and having a low degree of admixture confirmed by genome wide analysis [4]. They comprise approximately 17,384 individuals [5, 6], distributed in nine Indian Reservations, making them one of the largest indigenous groups in Brazil [7]. Traditionally hunter-gatherers, due to conflict with newcomer farmers, they started to be settled in delimited areas since 1957. After the contact and subsequent acculturation process, they became more sedentary, and modified their traditional diet by incorporating new foods, such as rice, sugar, and sweets [8]. Thus, important changes have been observed in the nutritional and health profile of this population, including diseases that were previously unknown to them, such as diabetes mellitus (DM) [9]. DM was first reported in the Xavante Indians in 1996 [10], when some members of the population sought medical assistance due to symptoms of metabolic decompensation and chronic DM complications [11].

The frequency of diabetic retinopathy (DR) among Xavante Indians is unknown. This survey aimed to describe the frequency of DR and potential risk factors for its development in this Brazilian Indian community.

## Methods

This study involved Xavante Indians from the Sangradouro/Volta Grande and São Marcos reservations located in Mato Grosso, Brazil. According to the Brazilian 2013 Census [6], the total population living in Sangradouro/Volta Grande was 882 individuals (623 adults ≥ 20 years of age) in 31 villages, and the total population in São Marcos was 3138 individuals (1588 adults ≥ 20 years of age) living in 28 villages. A previous population-based survey conducted from 2008 through 2012, including 948 Xavante Indians aged 20 years or more, disclosed 246 (25.9%) subjects with T2DM who were included in this study [12]. A non-probabilistic cluster sample out of the 246 subjects with DM was selected, that is, only those from the larger villages, were invited to participate. This procedure was adopted due to operational conditions and to optimize the field work. The study sample was composed by 140 individuals, being 40 men and 100 women. Non-participation was mainly due to the nomadic characteristics of this population, and cultural habits, like hunting, that keep a substantial portion of the population, especially males, out of their homes.

### Data collection

The collected information included the duration of DM, current DM treatment, smoking habits, anthropometric measurements (weight, height, waist circumference), blood pressure, capillary glycaemia (portable glucometer—*HemoCue*^*®*^ *Glucose201*^+^ *HemoCue AB, Angelholm, Sweden*), blood and urine samples. Blood and urine samples were separated in aliquots and stored at − 20 °C before transportation to the city of São Paulo for laboratory analyses. Diagnosis of DM was made if the individual had routine use of oral anti-diabetics or insulin, casual capillary glycemia ≥ 200 mg/dL, or a 2-h glucose level ≥ 200 mg/dL after a 75 g of glucose load, according to the World Health Organization (WHO) criteria [13]. The reference ranges used for laboratory tests were as follows: fasting glucose (abnormal ≥ 126 mg/dL); HbA1c (abnormal ≥ 6.5%); total cholesterol (abnormal ≥ 200 mg/dL); HDL cholesterol (abnormal < 40 mg/dL for men or < 50 mg/dL for women); LDL cholesterol (abnormal ≥ 100 mg/dL); triglycerides (abnormal ≥ 150 mg/dL); apolipoprotein A-I (abnormal < 120 mg/dL); apolipoprotein B (abnormal ≥ 120 mg/dL); C-reactive protein (abnormal ≥ 3.0 mg/dL); and microalbuminuria (MA) (abnormal ≥ 30 mg/L). The anthropometric measurements included weight, height, and waist circumference (abdominal circumference measured halfway between the edge of the last rib and the iliac crest). The body mass index (BMI) was expressed as kg/m^2^. Comparisons of indigenous people and Caucasians in North America have reported no difference in the relationships between visceral adipose tissue and BMI [14], total body fat [15], or waist circumference [16]. Therefore, we used the standard criteria for the reference values in this population involving waist circumference (abnormal ≥ 102 cm for men or ≥ 88 cm for women and BMI normal < 25; overweight ≥ 25 and < 30; obesity ≥ 30). Blood pressure was measured in the left arm using seated patients, after 5 min of rest, using the Omron HEM 742INTCH. It was measured three times and the average of the last two readings was reported. Hypertension was defined as systolic blood pressure (SBP) equal to or more than 140 mmHg and/or diastolic blood pressure (DBP) equal to or more than 90 mmHg, according to the WHO criteria [17].

The ophthalmic examination was performed twice, the first in 2009 and the second in 2011, and comprised measurement of LogMAR visual acuity, anterior segment biomicroscopy (*Kowa SL*-*15*^®^ portable slit-lamp), applanation tonometry (*Clement e Clarke*^®^ Perkins tonometer), and a detailed fundus examination under mydriasis with indirect ophthalmoscopy (*Keeler wireless*^®^ + 20D Aspheric Lens-*Volk*^®^). DR was graded according to the Early Treatment Diabetic Retinopathy Study (ETDRS), and categorised as follows: mild, moderate, severe, and very severe non-proliferative diabetic retinopathy (NPDR); early, high risk, and severe proliferative diabetic retinopathy (PDR) [18, 19].

### Statistical analysis

Student’s t tests were used to compare continuous clinical variables between two groups. Skewed variables were log transformed before these comparisons. Generalized linear regression with a log link and binomial distribution (log-binomial model) was used to assess the associations between clinical and demographic variables and the presence of retinopathy. These models are used to estimate crude and sex and age adjusted prevalence ratios (PR), with their associated 95% confidence intervals (95% CI). Associations were considered to be significant when 95% confidence intervals did not include 1 (similar to p < 0.05). The log-binomial models were fitted using the SAS software version 9.4.

The selection of a final subset of variables that best discriminate between individuals with and without DR used a conditional inference tree statistical modeling, a non-parametric class of multivariate regression model which embed tree-structured regression models into a well-defined theory of conditional inference procedures [20]. The use of conditional inference trees has several advantages in comparison with the traditional stepwise selection models, including: (a) conditional inference trees show details of the patterns of associations between the variables of interest, instead of only describing the variables that directly have associations with the dependent variable; and (b) continuous variables are directly inserted in the model and conditional inference procedures can define the optimal cut-off points for classifying individuals. The conditional inference trees were fitted with the package “party” of the software R version 3.4.3.

## Results

From the 140 T2DM subjects examined, 27 (19.3%) had clinical signs of DR in at least one eye. According to the ETDRS classification [19], most patients had non-proliferative retinopathy classified as mild in nine (33.3%), moderate in nine (33.3%), severe in six (22.3%), and very severe in two (7.4%) patients. Only one case (3.7%) of high risk proliferative DR was diagnosed. Indirect signs of diabetic macular edema were observed in four (14.8%) patients.

Table 1 shows clinical and laboratory findings of the patients with T2DM according to the presence or absence of retinopathy. The occurrence of DR was higher among those with a longer duration of diabetes (*p *< 0.01), and higher levels of triglycerides (p = 0.02), HbA1c (*p *< 0.01), glucose (*p *< 0.01) and microalbuminuria (p < 0.01).

**Table 1:** Diabetic retinopathy in Xavante Indians according to clinical and laboratory data

Variable	Diabetic retinopathy		*p* value^a^
	Yes (n = 27)	No (n = 113)	
	Mean (min–max)	Mean (min–max)	
Diabetes duration (months)	77.1 (12–180)	28.7 (0–132)	< 0.01
Fasting glucose (mg/dL)	356.4 (230–600)	219.7 (72–600)	< 0.01^b^
Total cholesterol (mg/dL)	162.5 (124–280)	148.4 (15–378)	0.06^b^
Triglycerides (mg/dL)	309.9 (88–3654)	220.3 (53–1981)	0.02^b^
HDL cholesterol (mg/dL)	40.0 (26.0–63.0)	39.3 (23.6–59.0)	0.63^b^
LDL cholesterol (mg/dL)	75.4 (10–140)	69.2 (27–116)	0.32
C-reactive protein (mg/L)	2.92 (0.48–41.44)	3.78 (0.35–83.05)	0.28^b^
HbA1c (%)	12.5 (8.2–16.0)	9.76 (5.5–18.8)	< 0.01^b^
Microalbuminuria (mg/L)	49.8 (2.2–778)	17.2 (1.1–5190)	< 0.01^b^
Apolipoprotein A–I (mg/dL)	114.1 (96.8–143)	110.7 (73–229.2)	0.23^b^
Apolipoprotein B (mg/dL)	71.4 (30.4–113)	75.2 (38–134)	0.42^b^

^a^Student’s t test for mean comparison

^b^Data were transformed in logarithmic values to a skewed distribution. In these cases, data are described by geometric means

Table 2 shows the clinical characteristics of the patients with T2DM according to the presence or absence of diabetic retinopathy, and the correspondent age and sex adjusted prevalence ratios (PR) obtained from the log-binomial model. PR for fasting glycaemia and HbA1c were not obtained due to the presence of a zero value in the cross-tabulation. The occurrence of DR was higher in the age-group 40–59-years (27.1%) and 60 or more years (24.3%) than in the 20–39 years (4.6%). The occurrence of DR was also higher among those with longer duration of DM diagnosis and papillary excavation ≥ 0.5. It was observed a non-significant crude association between DR and waist circumference (PR 1.7; 95% CI 0.8–3.4), but the sex and age adjusted PR indicates a significant association (PR 3.1; 95% CI 1.3–7.4). This difference between crude and adjusted PR suggests an interaction effect between waist circumference and sex or age groups. In fact, Table 3 shows that the association between waist circumference and DR is significant among women (PR 2.4; 95% CI 1.1–5.5), but this association is not significant among men (PR 1.7; 95% CI 0.3–7.6).

**Table 2:** Frequency of diabetic retinopathy in Brazilian Xavante Indians according to clinical data and age and sex adjusted prevalence ratios

	Total	Diabetic Retinopathy		Crude PR (95% CI)	Adjusted PR (95% CI)
		*n*	%		
Age (years)					
18–39	44	2	4.6	Reference	Reference
40–59	59	16	27.1	6.0 (1.4–24.6)*	6.3 (1.5–26.2)*
60 or more	37	9	24.3	5.3 (1.2–23.2)*	5.4 (1.2–23.5)*
Sex					
Men	40	7	17.5	Reference	Reference
Women	100	20	20.0	1.1 (0.5–2.5)	1.4 (0.6–3.0)
Duration of DM					
≤ 24 months	73	2	2.7	Reference	Reference
25–108 months	57	17	29.8	10.9 (2.6–45.2)*	2.3 (1.4–3.7)*
> 108 months	10	8	80.0	29.2 (7.2–118.6)*	3.8 (2.0–7.0)*
Smoking					
Yes	15	1	6.7	Reference	Reference
No	125	26	20.8	3.1 (0.4–21.4)	2.6 (0.3–17.7)
Waist circumference					
Abnormal	104	17	16.4	Reference	Reference
Normal	36	10	27.8	1.7 (0.8–3.4)	3.1 (1.3–7.4)*
BMI (kg/m^2^)					
< 25	19	6	31.6	Reference	Reference
25–29.9	51	13	25.5	0.8 (0.3–1.8)	0.5 (0.2–1.4)
≥ 30	70	8	11.4	0.4 (0.1–0.9)*	0.3 (0.1–0.8)*
Hypertension					
No	116	24	20.7	Reference	Reference
Yes	24	3	12.5	0.6 (0.1–1.8)	0.5 (0.1–1.5)
Cataract					
No	103	16	15.5	Reference	Reference
Yes	37	11	29.7	1.9 (0.9–3.7)	1.8 (0.8–4.1)
Papillary excavation					
0.1–0.4	128	21	16.4	Reference	Reference
0.5–0.9	12	6	50.0	3.0 (1.5–6.1)	2.7 (1.3–5.4)*
Visual acuity					
0–0.5	117	21	18.0	Reference	Reference
0.6–0.9	17	4	23.5	1.3 (0.5–3.4)	1.0 (0.3–2.7)
≥ 1	5	2	40.0	2.2 (0.7–7.0)	1.8 (0.6–5.3)
Fasting glycemia					
< 140 mg/dL	35	0	0	Reference	
140 mg/dL	105	27	25.7		
Total cholesterol					
< 200 mg/dL	121	25	20.7	Reference	Reference
≥ 200 mg/dL	15	2	13.3	0.6 (0.1–2.5)	0.7 (0.2–2.8)
HDL					
Normal	24	5	20.8	Reference	Reference
Abnormal	112	22	19.6	0.9 (0.3–2.2)	0.7 (0.2–1.7)
Triglycerides					
< 150 mg/dL	32	2	6.2	Reference	Reference
≥ 150 mg/dL	104	25	24.0	3.9 (0.9–15.3)	3.7 (0.9–14.7)
LDL					
< 100 mg/dL	101	18	17.8	Reference	Reference
≥ 100 mg/dL	14	2	14.3	0.8 (0.2–3.1)	0.8 (0.2–3.2)
C-reactive protein					
Normal	61	14	23.0	Reference	Reference
Abnormal	75	13	17.3	0.7 (0.3–1.5)	0.7 (0.3–1.4)
HbA1c (%)					
< 6.5	20	0	0	Reference	
≥ 6.5%	104	27	26.0		
Microalbuminuria					
Normal	76	13	17.1	Reference	Reference
Abnormal	45	14	31.1	1.8 (0.9–3.5)	1.9 (1.0–3.5)

*Significant at p < 0.05

**Table 3:** Association between waist circumference and DR stratified by sex

Sex	Waist circumference	Total	Diabetic Retinopathy		PR (95% CI)
			*n*	%	
Men	Abnormal	16	2	12.5	Reference
	Normal	24	5	20.8	1.7 (0.3–7.6)
Women	Abnormal	88	15	17.1	Reference
	Normal	12	5	41.7	2.4 (1.1–5.5)*

*Significant at p < 0.05

**Fig. 1:**
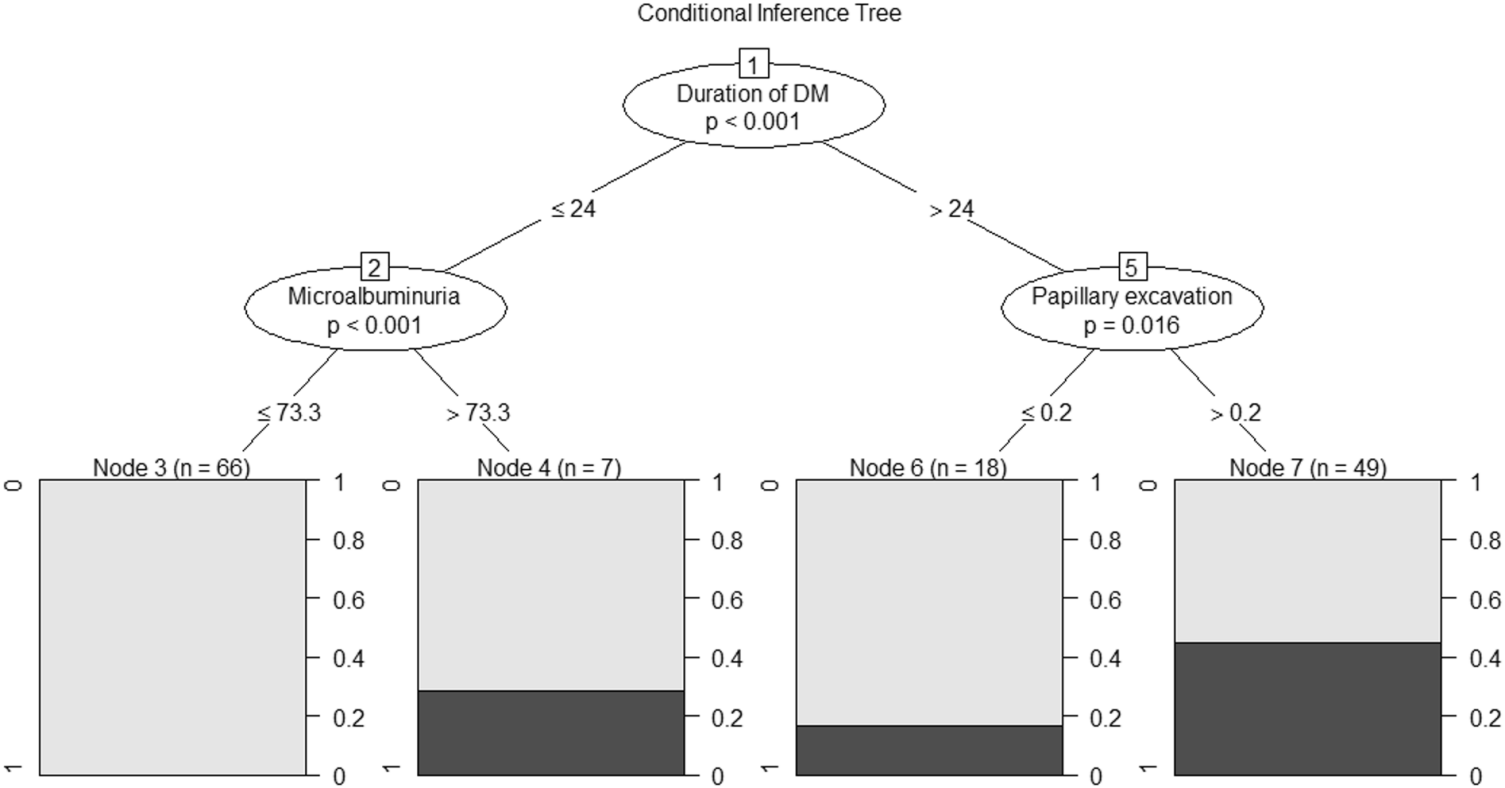
Conditional inference tree for the RD data. The rectangles at the bottom of the figure correspond to the “terminal nodes” and visually show the probabilities of DR for each path through the tree. The first node of the conditional inference tree (the “root node”) split the data according to the duration of DM (p < 0.001). Individuals with less than 24 months of DM were classified according to the MA values (interior node 2, p < 0.001), and those with values lower than 73.3 mg/L where classified as low risk for DR (terminal node 3). On the other hand, individuals with more than 24 months of DM were classified according to the papillary excavation (interior node 5, p = 0.016), where those with papillary excavation higher than 0.2 where classified as high risk for DR (terminal node 7). It is important to note that a cut-off value of 0.2 for the papillary excavation seems to be uninterpretable in a clinical point of view and it was obtained from an algorithm merely mathematical

Multivariate analysis was conducted using a conditional inference tree to find the best model of variables associated with DR. All the variables showed in Table 2 were used as split conditions in this analysis, but the computational algorithm selected the duration of DM, the microalbuminuria and the papillary excavation as those most able to jointly classify between individuals with and without DR (Fig. 1). For a better clinical interpretation, Table 4 shows the probabilities of DR considering the set of variables selected in the multivariate analysis, but considering the traditional reference ranges for each variable (MA values classified as abnormal if ≥ 30 mg/L, and papillary excavation classified according to the intervals 0.1–0.4 and 0.5–0.9).

**Table 4:** Association pattern between duration of DM, MA, papillary excavation and DR

Duration of DM	Total	Diabetic retinopathy	
		*n*	%
≤ 24 months			
Microalbuminuria normal	40	0	0
Microalbuminuria abnormal	17	2	11.8
> 24 months			
Papillary excavation 0.1–0.4	60	20	33.3
Papillary excavation 0.5–0.9	7	5	71.4

## Discussion

The Xavante Indians formed a genetically isolated population, with a low degree of miscegenation. The interaction between genetic susceptibility and changes in lifestyle is considered the main reason for the outbreak of a diabetes epidemic in this population [4, 12].

An early onset of T2DM (age of diagnosis < 40 years of age) has become increasingly prevalent, with a significant impact on the health care of these individuals. Obesity, a family history of T2DM, sedentary lifestyle, an ethnic minority, and female gender are some of the potential risk factors for this condition [21]. In an ongoing prospective cohort study in Asia, including 41,019 participants from nine countries, the overall rate of T2DM was 18% [22] versus 36.2% among the Xavante Indians [12]. Patients with early-onset T2DM are associated with an increased risk of premature development of microvascular and macrovascular complications [21]. The risk of developing premature retinopathy in these patients resulted predominantly from hypertension and prolonged exposure to suboptimal diabetes treatment [23].

A variety of techniques can be used to detect and classify diabetic retinopathy, including direct and indirect ophthalmoscopy, stereoscopic color film fundus photography, fluorescein angiography, and mydriatic or nonmydriatic digital color or monochromatic photography [24]. Ophthalmoscopy is the most commonly used technique to screen for diabetic retinopathy. However, has lower sensitivity relative to gold standard 7-field stereoscopic color photography as defined by the Early Treatment Diabetic Retinopathy Study (ETDRS) group [25].

The estimated overall prevalence of DR for 2010 in a systematic literature review conducted between 1980 and 2008 was 34.6% for any DR [26]. The current prevalence of DR in any Brazilian Indian population is unknown. In this study, the frequency of DR among Xavante Indians was 19.3%, lower than the reported prevalence of DR in the Brazilian population of 19.5–42.9% [27–31], which was lower than that in some indigenous populations in North America and Australia [32–35]. Table 1 shows that the average DM duration was 77.1 months in patients with DR versus only 28.7 months for subjects without DR. It is possible that the relatively short duration of the disease associated to lower sensitivity of fundoscopy to detect minimal changes such as microaneurysms, contributed to the lower rate of retinopathy in this population. Among Xavante Indians, the frequency of DR was significantly higher among those with DM duration ≥ 108 months than among those with duration less or equal to 24 months (adjusted PR = 3.8, 95% CI 2.0–7.0; Table 2).

The prevalence of diabetic macular edema (DME) is continuously rising worldwide and has become one of the major causes of vision loss in the working-age population. The gold standard in diagnosing DME still remains fluorescein angiography (FA). The optical coherence tomography (OCT) also can be used for screening, classification, monitoring, and treatment evaluation of DME [36]. Although fundoscopy does not allow adequate detection of DME, when compared to the methods described above, we found in this study four (14.8%) patients with clinical signs of macular edema.

Glycemic control is the most important independent risk factor for DR [37]. Several studies have identified an association between poor glucose control and an increased occurrence of DR [38–41]. In the present study, all 27 patients with DR had elevated levels of fasting glucose and HbA1c, reflecting poor metabolic control.

The diagnosis of glaucoma is traditionally based on the finding of optic nerve head (ONH) damage assessed subjectively by ophthalmoscopy or photography or by corresponding damage to the visual field assessed by automated perimetry, or both [42]. The stereoscopic optic nerve head photograph is the most accurate method for papillary excavation quantification and common imaging devices outperform most clinicians in classifying optic discs [43]. Although ophthalmoscopy is not the standard method for papillary excavation quantification, in this study, all patients who presented papillary excavation ≥ 0.5 and/or any other suspected alteration of the optic disc observed at ophthalmoscopy, were referred for investigation. Epidemiological studies have reported an association between DM and open-angle glaucoma [44–47]. Although this association is not fully understood, it is believed that DM causes microvascular flow impairment in the anterior portion of the optic nerve [48, 49]. In this study, it was not possible to determine which patients presented the diagnosis of glaucoma and whether there would be an association between this variable and DR. Despite this, the occurrence of DR was higher among those with papillary excavation ≥ 0.5 (adjusted PR = 2.7, 95% CI 1.3–5.4; Table 2).

Hypertension is a very common comorbidity in DM, affecting approximately 20–60% of these patients [50]. A previous study reported that the prevalence of hypertension among Xavante Indians was 17.5% [12], which was lower than the 20% found in the general adult Brazilian population [51]. Several studies have reported an association of hypertension with DR [32, 52–55]. Despite the report of this association, it was not found among the Xavante Indians (adjusted PR = 0.5, 95% CI 0.1–1.5; Table 2). However, this finding needs to be confirmed because the small sample size may have biased the results.

Although there are reports of an association of DR with other variables such increased levels of total cholesterol [56] and triglycerides [57], we could not find association of total cholesterol or its fractions with DR in the Xavante population, but individuals with DR had mean values of triglycerides higher than individuals without the disease (p = 0.02; Table 1).

Diabetic nephropathy develops in approximately 20–40% of type 1 diabetic patients and in less than 20% of type 2 diabetic patients [58]. Past studies have reported an association between DR and MA [59, 60]. In the present study, we found 14 patients who developed MA and diabetic retinopathy, 13 patients who developed retinopathy but not MA, and 33 patients who developed only MA. Initially, we found no association between MA and diabetic retinopathy (adjusted PR = 1.9, 95% CI 1.0–3.5; Table 2). However, the multivariate analysis using a conditional inference tree evidenced that MA is statistically associated with DR only for the Xavante Indians with less than 24 months of DM (p < 0.001, Fig. 1).

There are limitations to this study. A major limitation was low participation in the study (56%), which may have biased some of the results. Individuals from small villages were not included in the ophthalmologic survey to optimize the field work carried out in two visits. The lower sensitivity of fundoscopy to detect minimal changes may also have contributed to the lower rate of retinopathy in this population. The small number of cases (*n* = 27) also reduced the power to detect associations. The poor diabetes control for a prolonged period of time may have influenced the relationship between some variables as weight excess, waist circumference, sex and DR. The presence of DR is also an indicator that other chronic diabetes complications are being developed.

## Conclusion

The presence of DR (19.3%) in Xavante Indians is an alert for health care providers for this population, since diabetes is a new disease among them. Its association with disease duration, high levels of HbA1c and blood glucose calls attention for the necessity of more actions to improve diabetes control in this recently contacted ethnic group that needs particular attention.

## Abbreviations

DR: diabetic retinopathy
HbA1c: glycated hemoglobin
DM: diabetes mellitus
T2DM: type 2 diabetes mellitus
MA: microalbuminuria
BMI: body mass index
SBP: systolic blood pressure
DBP: diastolic blood pressure
WHO: World Health Organization
ETDRS: Early Treatment Diabetic Retinopathy Study
NPDR: non-proliferative diabetic retinopathy
PDR: proliferative diabetic retinopathy
DME: diabetic macular edema
FA: fluorescein angiography
OCT: optical coherence tomography
ONH: optic nerve head
